# Hitting a Wall: An Ambiguous Case of Wallenberg Syndrome

**DOI:** 10.7759/cureus.16268

**Published:** 2021-07-08

**Authors:** James R Pellegrini, Rezwan Munshi, Bohao Cao, Samuel Olson, Vincent Cappello

**Affiliations:** 1 Internal Medicine, Nassau University Medical Center, East Meadow, USA; 2 Physical Medicine and Rehabilitation, Stony Brook University, Stony Brook, USA

**Keywords:** wallenberg syndrome, neurology, ambiguous, internal medicine, mri

## Abstract

Wallenberg syndrome is the most common stroke of the posterior circulation. Diagnosis of Wallenberg syndrome is often overlooked as initial MRI may show no visible lesion. We present an atypical case of Wallenberg syndrome in which the initial MRI of the brain was normal.

Our patient is a 65-year-old male who was brought in by emergency medical services complaining of right-sided facial droop, slurred speech, and left-sided weakness for one day. Physical examination showed decreased left arm and leg strength compared to the right side, decreased left facial temperature sensations, decreased left arm and leg temperature sensations, and difficulty sitting upright with an associated leaning towards the left side. An initial magnetic resonance imaging (MRI) of the brain with and without contrast revealed no abnormality. In light of such a high suspicion for stroke based on the patient’s neurologic deficits, a repeat MRI of the brain was performed three days later and exposed a small focus of bright signal (hyperintensity) on T2-weighted fluid-attenuated inversion recovery and diffusion-weighted imaging (DWI) in the left posterior medulla.

Wallenberg syndrome, also known as lateral medullary syndrome or posterior inferior cerebellar artery syndrome, is a constellation of symptoms caused by posterior vascular accidents. The neurological deficits associated with this disease are due to damage of the lateral medulla, inferior cerebellar peduncle, nucleus of trigeminal nerve, nucleus and fibers of vagus and glossopharyngeal nerves, descending sympathetic tract, spinothalamic tract, and/or vestibular nuclei. MRI with DWI is the gold standard to confirm the diagnosis.

Wallenberg syndrome has the potential to leave patients extremely debilitated. Early detection, management, and rehabilitation are critical for improving post-stroke recovery.

## Introduction

Approximately 20% of all strokes that occur annually in the United States are located in the posterior circulation [1]. Wallenberg syndrome happens to be the most common stroke of the posterior circulation [1]. It is a collection of neurologic deficits caused by posterior vascular accidents at the level of the posterior medulla. Diagnosis of Wallenberg syndrome is often overlooked as MRI has been shown to be normal in up to 30% of cases [1]. We present an atypical case of Wallenberg syndrome in which the initial MRI of the brain was normal.

## Case presentation

Our patient is a 65-year-old male with a medical history of hypertension and hyperlipidemia, who was brought in by emergency medical services complaining of right-sided facial droop, slurred speech, and left-sided weakness of one-day duration. The patient reported that he returned home from breakfast one day ago, sat on the front steps of his home, and suddenly felt dizzy and "passed out." It was unknown how long he lost consciousness as it was unwitnessed. He denied a prior history of stroke or myocardial infarction. The rest of the review of systems was unremarkable.

Physical examination showed decreased left arm and leg strength compared to the right side, decreased left facial temperature sensations, decreased left arm and leg temperature sensations, and difficulty sitting upright with an associated leaning towards the left side. Vision tests were difficult to assess and compare as he had a severe right cataract.

Initial laboratory analysis was remarkable for an elevated creatinine of 1.4 mg/dL (normal 0.7-1.3 mg/dL), elevated troponin I high sensitivity of 54.20 ng/L (normal 0-53.53 ng/L), and thyroid-stimulating hormone of 0.14 uIU/mL (normal 0.55-4.78 uIU/mL). Urinalysis revealed moderate leukocyte esterase, positive nitrites, and urine white blood cells greater than 50. Urine culture grew *Escherichia coli* with a colony count greater than 100,000 CFU/mL and was susceptible to nitrofurantoin.

**Figure 1:**
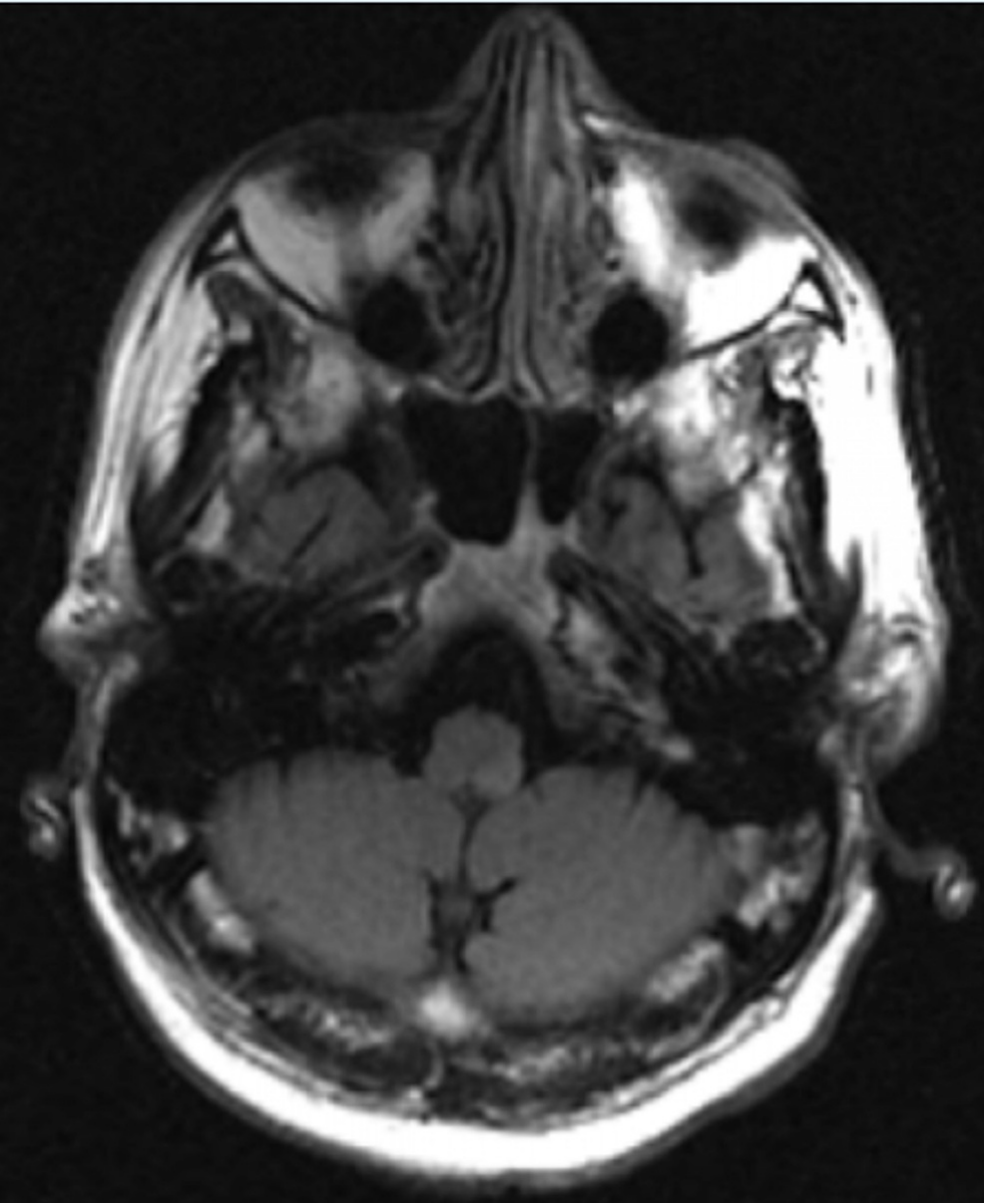
Initial MRI of the brain showing no significant hyperintensity on T2-FLAIR T2-FLAIR: T2-weighted fluid-attenuated inversion recovery

**Figure 2:**
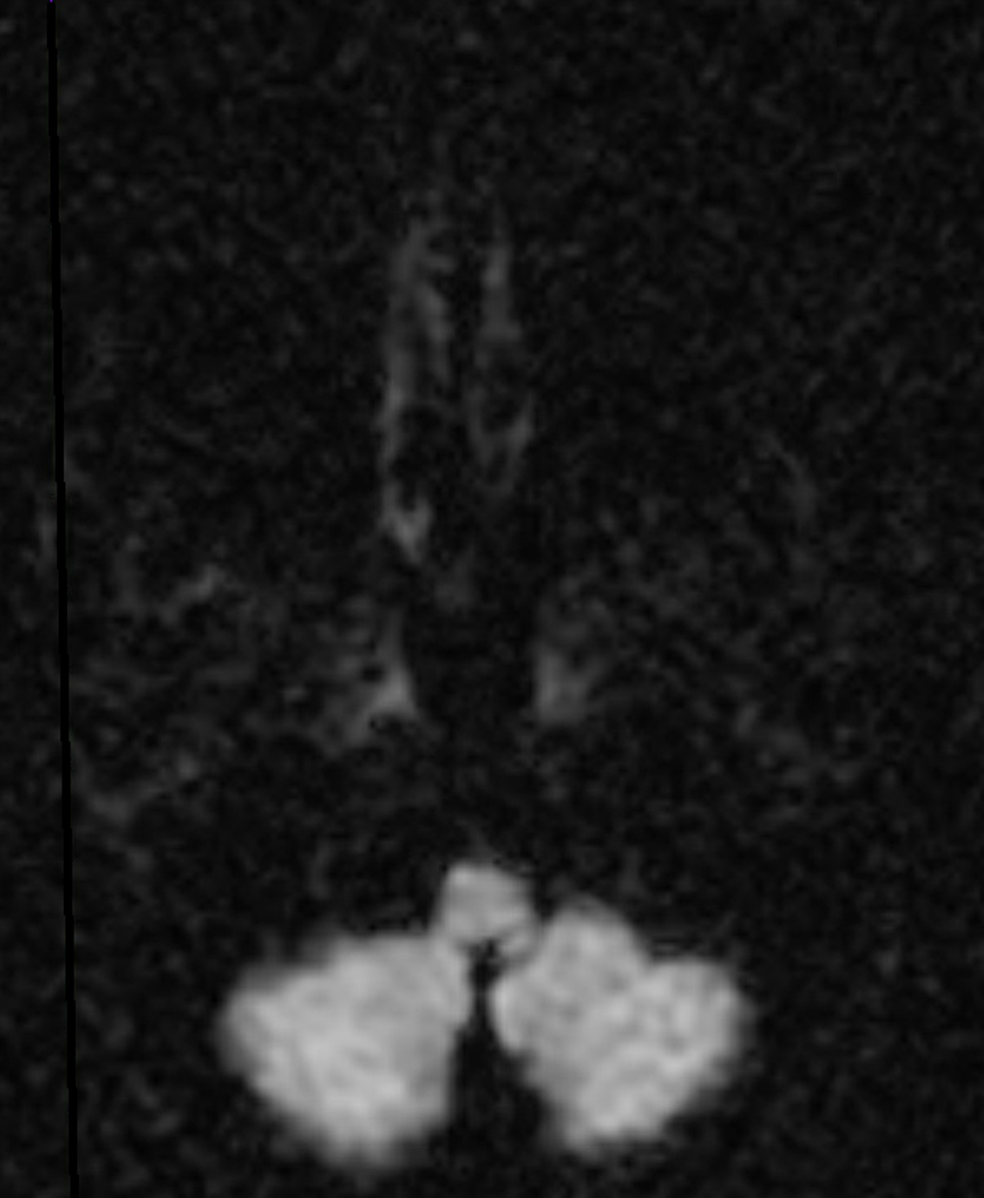
Initial MRI of the brain showing no significant hyperintensity on DWI DWI: diffusion-weighted imaging

**Figure 3:**
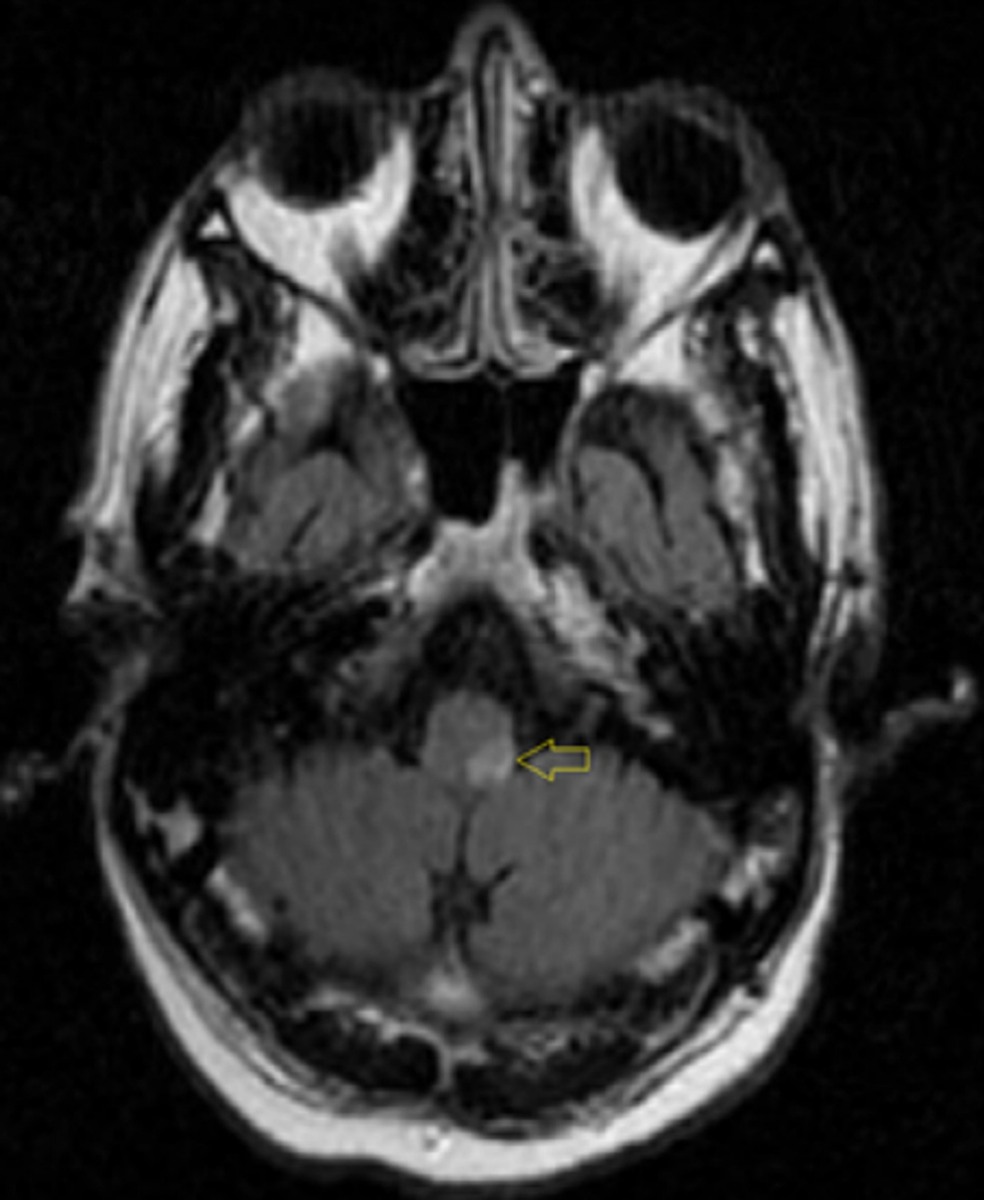
MRI of the brain showing a small focus of bright signal (hyperintensity) on T2-FLAIR T2-FLAIR: T2-weighted fluid-attenuated inversion recovery

**Figure 4:**
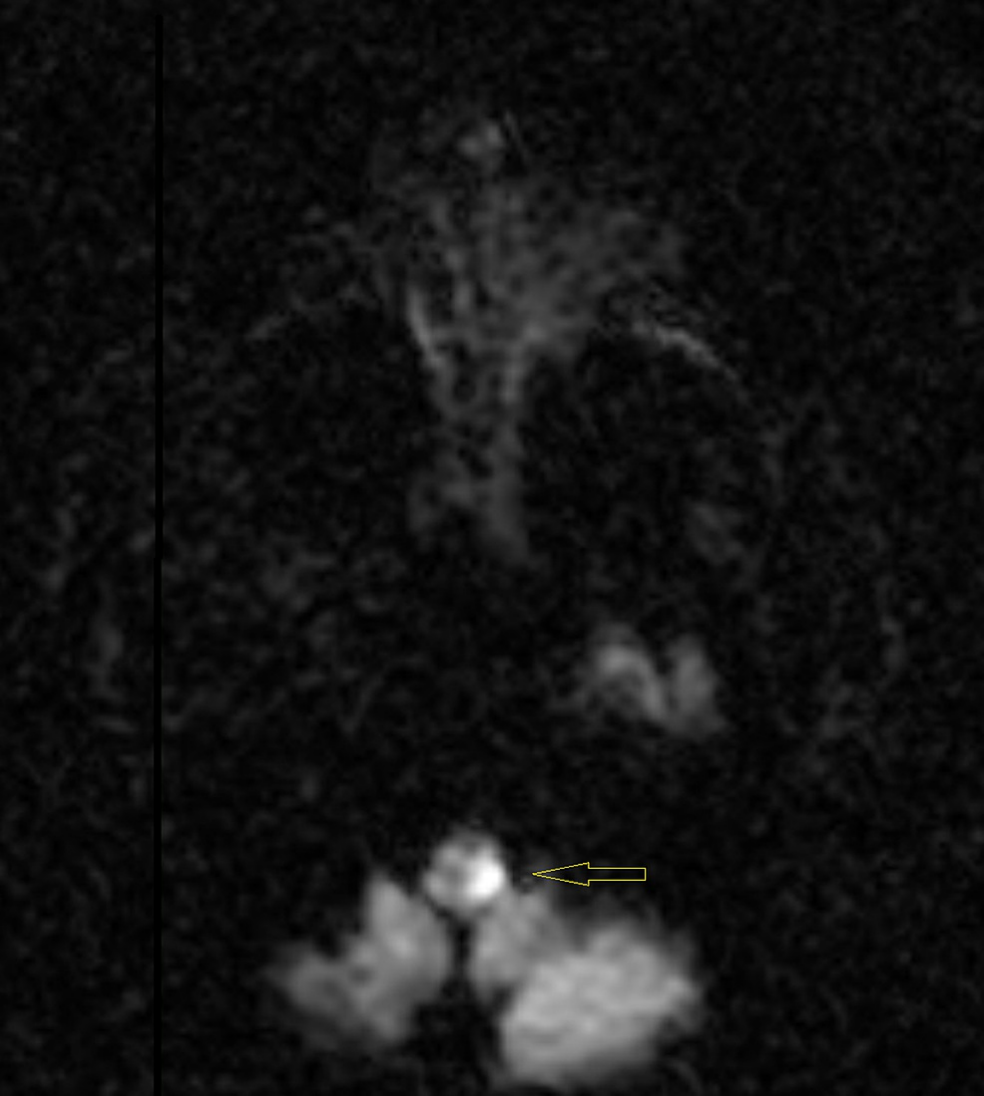
MRI of the brain showing a small focus of bright signal (hyperintensity) on DWI DWI: diffusion-weighted imaging

A computed tomography (CT) scan of the head showed no acute intracranial pathology. A CT angiography (CTA) of the head and neck showed no significant stenosis, aneurysm, or vascular malformation. An echocardiogram was performed and showed a left ventricular ejection fraction of 65% to 70%, normal left ventricular size and systolic function, and no segmental wall motion abnormalities. An initial magnetic resonance imaging (MRI) of the brain with and without contrast revealed no abnormality (Figures 1, 2). In light of such a high suspicion for stroke based on the patient’s neurologic deficits, a repeat MRI of the brain was performed three days later and exposed a small focus of bright signal (hyperintensity) on T2-weighted fluid-attenuated inversion recovery (T2-FLAIR) (Figure 3) and diffusion-weighted imaging (DWI) in the left posterior medulla (Figure 4). The patient was started on antibiotics for his urinary tract infection, high-intensity statin therapy, low-dose aspirin therapy, and eventually discharged to subacute rehabilitation for further management once medically stabilized.

## Discussion

Wallenberg syndrome, also known as lateral medullary syndrome or posterior inferior cerebellar artery syndrome, is a constellation of symptoms caused by posterior vascular accidents. It is the most common form of all clinical posterior ischemic infarctions and arises predominantly in men during their sixth decade [1,2].

The pathogenesis of Wallenberg syndrome is presumably caused by large vessel infarction (50%), followed by arterial dissection (15%), small vessel infarction (13%), and cardiac embolism (5%). Of interest, a clinical-radiology study showed that vertebral artery disease, which gives branches to the posterior inferior cerebellar artery (PICA) and the anterior spinal artery, is the most common cause of Wallenberg syndrome (67%), and only a small percentage of the disease has PICA involvement (10%) [3]. The most prevalent risk factors for arterial atherothrombotic occlusion include hypertension, smoking, and diabetes. In a young patient, vertebral artery dissection - potentially due to neck injuries/manipulation, Ehlers Danlos syndrome, or Marfan syndrome - should be considered [4].

Diagnosis of Wallenberg syndrome is made clinically and diagnostically based on presenting symptoms, history of presentation, and radiographic imaging. The neurological deficits associated with this disease are due to damage of the lateral medulla, inferior cerebellar peduncle, nucleus of trigeminal nerve, nucleus and fibers of the vagus and glossopharyngeal nerves, descending sympathetic tract, spinothalamic tract, and/or vestibular nuclei [5,6]. The onset of the disease is acute. The most common symptoms are dizziness with vertigo, nystagmus, gait instability, hoarseness of voice, and dysphagia [7]. Other deficits can be found in combinations depending on the extent of the infarct, including, but not limited to, feeling of being pulled towards the side of the lesion when walking and difficulty sitting upright without support, blurry vision, diplopia, ipsilateral Horner’s syndrome (triad of miosis, ptosis, and anhidrosis), and autonomic dysregulation due to lesion of the descending sympathetic tract [8]. Other neurologic deficits include pain (i.e., sharp, dull, or burning) on the ipsilateral side of the face that is not related to other causes, ipsilateral decreased pain and temperature sensations in the face with possible decrease of corneal reflex, and contralateral decreased pain and temperature sensations in the upper and lower extremities. Hiccups, uvula deviation towards the contralateral side of the lesion, and hypotonia of the ipsilateral arm - demonstrated by having the patient quickly raising and lowering both of their outstretched hands (the symptomatic arm will overshoot upon braking compared to the other arm) - may also be found in Wallenberg syndrome.

CT scan should be done initially to rule out the possibility of a hemorrhagic stroke. Once a hemorrhagic stroke is ruled out, an MRI should be obtained to gather a more detailed image of the brain. MRI with DWI is the gold standard to confirm the diagnosis [9]. However, it is noted that up to 30% of cases do not have a lesion present on MRI with DWI, which often leads to misdiagnosis of this disease [10].

Management of Wallenberg syndrome involves treating the patient for an acute ischemic stroke. Intravenous (IV) thrombolytic therapy such as tissue plasminogen activator (tPA) is indicated if the onset of symptoms is within 3-4.5 hours of presentation to the hospital [11]. Permissive hypertension (HTN) should be allowed for 24-48 hours unless systolic blood pressure (SBP) is above 220 mmHg or diastolic blood pressure is above 120 mmHg [12]. If tPA is given, then blood pressure should be maintained below 180/105 [12]. Workup involving an echocardiogram, CT angiography of the head and neck, as well as lipid panel, thyroid-stimulating hormone, hemoglobin A1C, erythrocyte sedimentation rate, C-reactive protein, and blood cultures should be obtained to assess for etiology and modifiable risk factors of the stroke [12]. Speech and swallow assessment should be made to assess a patient's swallowing capabilities and prevent the possibility of aspirating. A medication regimen involving aspirin 81 mg daily and lipid-lowering medication should be given as secondary stroke preventative measures [12]. Smoking cessation, diabetes control, blood pressure control, and healthy diet and exercise are also vital in preventing a recurring stroke [8].

The overall prognosis of Wallenberg syndrome is better than most other acute ischemic strokes. However, like all stroke syndromes, permanent disability may occur. The most common sequela of Wallenberg syndrome is gait instability. Early physical and occupational therapy with a good rehabilitation plan is vital for improving post-stroke recovery.

Our patient presented with severe dizziness and vertigo, left-sided facial burning sensation, and intractable hiccups upon admission. Physical examination showed decreased left arm and leg strength compared to the right side, decreased left facial temperature sensations, decreased left arm and leg temperature sensations, and difficulty sitting upright with an associated leaning towards the left side, indicating neurologic deficits consistent with Wallenberg syndrome. The MRI of the brain showed a lateral, hyperintense lesion at the level of the left posterior medulla, which confirmed the proper location for the diagnosis of Wallenberg syndrome.

## Conclusions

Wallenberg syndrome is a constellation of symptoms caused by posterior vascular accidents. MRI with DWI is the gold standard to confirm the diagnosis. We present a rare case of Wallenberg syndrome in a patient who initially had a normal MRI, but the repeat MRI showed a lateral, hyperintense lesion at the level of the left posterior medulla. Even though Wallenberg syndrome is often misdiagnosed due to a normal MRI, it is imperative to still have it in the differential diagnosis as early management and rehabilitation can be life-saving.

## References

[1] Lui F, Tadi P, Anilkumar AC (2021). Wallenberg syndrome. StatPearls [Internet].

[2] Saleem F, M Das J (2020). Lateral medullary syndrome. StatPearls [Internet].

[3] Kim JS (2003). Pure lateral medullary infarction: clinical-radiological correlation of 130 acute, consecutive patients. Brain.

[4] Park MG, Choi JH, Yang TI, Oh SJ, Baik SK, Park KP (2014). Spontaneous isolated posterior inferior cerebellar artery dissection: rare but underdiagnosed cause of ischemic stroke. J Stroke Cerebrovasc Dis.

[5] Kim H, Lee HJ, Park JW (2018). Clinical course and outcome in patients with severe dysphagia after lateral medullary syndrome. Ther Adv Neurol Disord.

[6] Battel I, Koch I, Biddau F (2017). Efficacy of botulinum toxin type-A and swallowing treatment for oropharyngeal dysphagia recovery in a patient with lateral medullary syndrome. Eur J Phys Rehabil Med.

[7] Searls DE, Pazdera L, Korbel E, Vysata O, Caplan LR (2012). Symptoms and signs of posterior circulation ischemia in the new England medical center posterior circulation registry. Arch Neurol.

[8] Caplan L (2000). Posterior circulation ischemia: then, now, and tomorrow. The Thomas Willis Lecture-2000. Stroke.

[9] De Cocker LJ, Lövblad KO, Hendrikse J (2017). MRI of cerebellar infarction. Eur Neurol.

[10] Makin SD, Doubal FN, Dennis MS, Wardlaw JM (2015). Clinically confirmed stroke with negative diffusion-weighted imaging magnetic resonance imaging: longitudinal study of clinical outcomes, stroke recurrence, and systematic review. Stroke.

[11] Salerno A, Cotter BV, Winters ME (2017). The use of tissue plasminogen activator in the treatment of Wallenberg syndrome caused by vertebral artery dissection. J Emerg Med.

[12] Sabatine MS (2020). Neurology: Stroke. Pocket Medicine: The Massachusetts General Hospital Handbook of Internal Medicine.

